# Correlation between Cardiovascular Diseases and Tooth Loss in the Brazilian Population: an eco-infodemiology study

**DOI:** 10.21142/2523-2754-1404-2026-315

**Published:** 2026-10-01

**Authors:** Rodrigo Könsgen Rossales, Gabriel Massami Bernardes Utiyama, Lucas Saraiva Mascarenhas, Alexandre Santos de Oliveira, Jessica de Vargas Bosenbecker, Shana Oliveira Machado Kolás, Francisco Wilker Mustafa Gomes Muniz, Thiago Marchi Martins

**Affiliations:** 1 Graduation Program in Dentistry, Federal University of Pelotas. Pelotas, Brazil. rodrigokonsgen@gmail.com shaomkolas@gmail.com Graduation Program in Dentistry Federal University of Pelotas Pelotas Brazil rodrigokonsgen@gmail.com shaomkolas@gmail.com; 2 School of Dentistry, Federal University of Pelotas. Pelotas, Brazil. gabrielmbu15@gmail.com lucasmascarenhas2004@gmail.com santosxandinho93@gmail.com jessicabosenbecker@gmail.com School of Dentistry Federal University of Pelotas Pelotas Brazil gabrielmbu15@gmail.com lucasmascarenhas2004@gmail.com santosxandinho93@gmail.com jessicabosenbecker@gmail.com; 3 Semiology and Clinic Department, Federal University of Pelotas. Pelotas, Brazil. wilkermustafa@gmail.com thiagoperio@yahoo.com.br Semiology and Clinic Department Federal University of Pelotas Pelotas Brazil wilkermustafa@gmail.com thiagoperio@yahoo.com.br

**Keywords:** tooth loss, heart, ecological study, edentulous mouth, pérdida dental, corazón, estudio ecológico, boca edéntula

## Abstract

**Objective:**

Chronic inflammation is a shared pathway linking oral diseases and cardiovascular diseases (CVD), and tooth loss has been proposed as a marker of cumulative inflammatory burden. This eco-infodemiological study aimed to evaluate correlations between self-reported CVD and tooth loss in Brazilian population.

**Materials and Methods:**

Data from the 2019 National Health Survey (“Pesquisa Nacional de Saúde” - PNS) was collected. Pearson correlation was used to assess correlation between cardiovascular outcomes and two exposure definitions: adults who lost all teeth and adults who lost ≥13 teeth. The Brazilian states were considered the unit of analysis**.**

**Results:**

Mild to moderate and significant correlations were found between total edentulism and CVD with functional limitations (R = 0.533; p = 0.004) and between total edentulism and CVD with prior surgery (R = 0.392; p = 0.043). Tooth loss of ≥13 teeth also correlated with CVD with functional limitations (R = 0.388; p = 0.046), although no significant association emerged for CVD with surgery (R = 0.311; p = 0.114). For broader CVD (without limitations or surgery), significant correlations persisted for total edentulism (R = 0.403; p = 0.037), but not for ≥13 teeth (R = 0.345; p = 0.078). These patterns suggest that greater cumulative oral disease burden is moderately correlated with more severe CVD.

**Conclusions:**

Altogether, the findings support tooth loss, especially edentulism, as a potential marker of systemic vulnerability, due to the chronic inflammation process involved.

## Resumen

**Objetivo:**

La inflamación crónica es una vía común que vincula las enfermedades bucales con las enfermedades cardiovasculares (ECV) y la pérdida dental se ha propuesto como un marcador de la carga inflamatoria acumulada. Este estudio ecoinfodemiológico tuvo como objetivo evaluar las correlaciones entre las ECV autoinformadas y la pérdida dental en la población brasileña.

**Materiales y métodos:**

: Se recopilaron datos de la Encuesta Nacional de Salud de 2019 (Pesquisa Nacional de Saúde - PNS). Se utilizó la correlación de Pearson para evaluar la correlación entre los resultados cardiovasculares y dos definiciones de exposición: adultos que perdieron todos los dientes y adultos que perdieron ≥13 dientes. Los estados brasileños se consideraron la unidad de análisis.

**Resultados:**

Se encontraron correlaciones leves a moderadas y significativas entre el edentulismo total y las ECV con limitaciones funcionales (R = 0,533; p = 0,004), así como entre el edentulismo total y las ECV con cirugía previa (R = 0,392; p = 0,043). La pérdida de ≥ 13 dientes también se correlacionó con la enfermedad cardiovascular (ECV) con limitaciones funcionales (R = 0,388; p = 0,046), aunque no se observó una asociación significativa con la ECV asociada a cirugía (R = 0,311; p = 0,114). En la ECV en general (sin limitaciones ni cirugía) persistieron correlaciones significativas con el edentulismo total (R = 0,403; p = 0,037), pero no con ≥ 13 dientes (R = 0,345; p = 0,078). Estos patrones sugieren que una mayor carga acumulada de enfermedad oral se correlaciona de manera moderada con una ECV más grave.

**Conclusiones:**

En conjunto, los hallazgos respaldan la pérdida dental, especialmente el edentulismo, como un posible marcador de vulnerabilidad sistémica, debido al proceso de inflamación crónica involucrado.

## INTRODUCTION

Cardiovascular diseases, such as heart attacks, strokes, and heart failure, are intrinsically linked to several systemic factors, one of which is chronic inflammation. These conditions represent one of the leading causes of morbidity and mortality worldwide and are influenced by a complex interaction of genetic, behavioral, and environmental factors ^1^. Cardiovascular diseases can be caused or exacerbated by several factors and habits, among which smoking is one of the main contributors. A review explored the mechanisms by which smoking contributes to the development of cardiovascular diseases, demonstrating that it is associated with inflammation, blood coagulation, endothelial dysfunction, and the development of atherosclerosis ^2^.

Chronic inflammation plays a central role in the relationship with oral diseases and other systemic diseases. Diabetes and cardiovascular diseases have also been shown to be major mechanisms involved in the progression of these pathological conditions, which negatively impact patients’ lives ^3^. Oral health is recognized as an essential component of quality of life, and oral diseases, such as dental caries, gingivitis, and periodontitis, constitute a global health problem. In 2010, untreated dental caries was the most prevalent disease worldwide ^4^.

Dental caries and periodontitis are among the most common diseases in humans and are the leading causes of tooth loss. Both conditions can impact patients’ self-esteem and quality of life and are associated with inflammatory states-periodontitis and gingivitis through direct inflammation, and dental caries through the development of odontogenic abscesses ^5^. Regarding gingivitis and periodontitis, inflammation has been recognized as a central component in the pathogenesis of these conditions ^6^^,^^7^. Understanding the relationship between cardiovascular diseases and oral conditions related to tooth loss is crucial to comprehending the complex interactions that affect both oral and systemic health ^8^.

Bacteria from the oral cavity-particularly periodontal pathogens-can translocate into the bloodstream, triggering a systemic inflammatory response ^9^. Diabetes mellitus is a chronic metabolic disease related to persistent inflammation and presents a bidirectional relationship with oral inflammation, increasing susceptibility to infections and impairing wound healing, including periodontal tissues. Moreover, both oral diseases and diabetes are independent risk factors for the development of cardiovascular diseases. Shared mechanisms such as systemic inflammation, endothelial dysfunction, and atherosclerotic plaque formation are strongly associated with these conditions ^10^.

Oral diseases such as odontogenic abscesses, gingivitis, periodontitis, and others exert a substantial negative impact on oral health-related quality of life (OHRQoL) ^11^. Inflammatory processes have a strong influence on individuals' overall health and are associated with factors that lead to tooth loss, which may culminate in edentulism as a consequence of cumulative ablative events throughout life, primarily arising from a clinical approach centered on dental extractions ^12^.

However, social inequality can result in disparities in tooth loss, as individuals with lower income tend to have reduced access to dental care, increasing the risk of losing teeth ^13^. Additionally, income disparities significantly influence dietary quality, as low-income individuals tend to consume poorer-quality diets, which can increase the risk of both diabetes and cardiovascular diseases ^14^^,^^15^.

When considering edentulism, it is common to associate it predominantly with dental caries and, to a lesser extent, with periodontitis. However, the inflammatory state triggered in the body by the oral disease that led to tooth loss may negatively influence overall health. Thus, the literature suggests a possible correlation between tooth loss and cardiovascular diseases. Studies have reported a potential causal link between edentulism and the incidence of cardiovascular diseases in adult populations, particularly in older individuals ^16^.

Therefore, the aim of this study was to conduct an eco-infodemiological study, a novel approach to explore public data available data ^17^, to explore whether there is a correlation between cardiovascular diseases and self-reported tooth loss (partial or total edentulism) among Brazilian adults. Based on the factors described above, the hypothesis is that there is a correlation between self-reported cardiovascular diseases and tooth loss (total or partial edentulism) in the Brazilian population.

## MATERIALS AND METHODS

### Study design

This eco-infodemiological study sought to identify the correlation between individuals who report a medical diagnosis of cardiovascular diseases and tooth loss in Brazil, using an in-depth bibliographic approach based on data from the Brazilian Institute of Geography and Statistics (Instituto Brasileiro de Geografia e Estatística - IBGE). Consequently, ethical review by a research ethics committee was not required.

Additionally, publicly accessible population data were considered to identify potential factors that may intensify this correlation, such as level of education, age, income, sex, macro-region (place of residence), and skin color. To maintain alignment with the most recent national context, the 2022 Brazilian census (IBGE, 2023) was used as a reference.

### Data collection

All information was obtained from the IBGE Automatic Recovery System (Sistema IBGE de Recuperação Automática - SIDRA), referring to the 2019 National Health Survey (Pesquisa Nacional de Saúde - PNS), a nationwide population-based survey conducted by the IBGE in partnership with the Brazilian Ministry of Health. It collects comprehensive information on health conditions, the prevalence of chronic diseases, lifestyle factors, and the population’s access to and use of health services in Brazil. Data related to cardiovascular diseases and tooth loss were collected and categorized into the following subgroups:

### Cardiovascular disease outcomes:

- Adults (≥18 years) reporting a medical diagnosis of heart disease with intense or very intense limitations in daily activities due to the condition or its complications;
- Adults reporting a medical diagnosis of heart disease who have undergone coronary artery bypass surgery, stent placement, or angioplasty;
- Adults reporting any medical diagnosis of heart disease.

### Tooth loss exposures:

Adults (≥18 years) who lost all teeth;

Adults (≥18 years) who lost 13 or more teeth.

To all collected variables, the unit of analyses was the Brazilian states and the Federal District. The variables collected were the percentage of sex, income, education, skin color, and age in each Brazilian state. Additional data on the Human Development Index (2021) (“Painel IDHM | United Nations Development Programme”), oral health coverage in primary care in 2022 ^18^^,^^19^, and the Gini Index in 2021 ^20^ were also obtained, categorized by Brazilian states. Data collection was performed collaboratively by all researchers and compiled into a Microsoft Excel spreadsheet.

Self-reported tooth loss and cardiovascular disease data were analyzed in relation to one another and presented categorically. Age was divided into three groups for analysis: 18-29 years, 30-59 years, and ≥60 years, due to differences in how outcomes are presented in SIDRA.

### Statistical analysis

All analyses were performed using IBM SPSS Statistics, version 26. Shapiro-Wilk was used to assess data distribution. Pearson correlation test was used to assess correlation between outcomes and exploratory variables, as symmetrical distributions were detected in all outcomes (CD with limitations [p = 0.145], CD submitted to surgery [p = 0.514], CD without limitations or submitted to surgery [p = 0.669]). A p-value < 0.05 was considered statistically significant.

## RESULTS

Table 1 shows a consistent pattern in which greater tooth loss is correlated with cardiovascular outcomes, particularly among adults with functional limitations. Significant, but weak to moderate, correlations were observed for individuals with 13 or more missing teeth and for those who were completely edentulous, including participants who had undergone surgery. Although some comparisons in groups without limitations did not reach statistical significance, the overall trend suggests that increasing severity of tooth loss is accompanied by a higher correlation for cardiovascular conditions.

**Table 1:** Correlation between cardiovascular diseases outcomes and teeth loss exposures

	R value	P-value
CD with limitations X Adults who lost 13 or more teeth	0.388*	0.046*
CD with limitations X Adults who lost all teeth	0.533*	0.004*
CD submitted to surgery X Adults who lost 13 or more teeth	0.311*	0.114*
CD submitted to surgery X Adults who lost all teeth	0.392	0.043*
CD without limitations or submitted to surgery X Adults who lost 13 or more teeth	0.345*	0.078*
CD without limitations or submitted to surgery X Adults who lost all teeth	0.403*	0.037*

CD - Cardiovascular diseases; * Pearson’s Correlation. Bold values mean statistically significant correlation (p < 0.05).

It was identified a moderate correlation between HDI and self-reported cardiovascular disease without limitation or surgery (R = 0.716, P < 0.001), cardiovascular disease with limitation (R = 0.681, p < 0.001), and cardiovascular disease with surgery (R = 0.739, P < 0.001). Fair correlation was found between tooth loss exposures and HDI (R = 0.163; P = 0.416 for adults who lost all teeth and R = -0.112; P = 0.579 for adults who lost 13 or more teeth).

There was a fair and inverse correlation between Gini’s index and self-reported cardiovascular disease without limitation or surgery (R = -0.444, P = 0.020), and cardiovascular disease with limitation (R = -0.493, P = 0.009), but not with cardiovascular disease with surgery (R = -0.445, P = 0.020). Significant correlations were also not found between adults who lost all teeth (R = -0.202, P = 0.313), but moderate correlation was found between adults who lost 13 or more teeth (R = -0.058, P = 0.773) with Gini’s Index. 

Significant correlation was found between adults who lost 13 or more teeth and oral health coverage in primary health care (R = 0.449, P = 0.019). Despite that, no significant correlation was found between adults who lost all teeth and oral health coverage in primary health care (R = 0.298, P = 0.131).

## DISCUSSION

The results of this study highlight significant correlations between tooth loss and many characteristics of cardiovascular diseases among the Brazilian states, which is consistent with previous evidence suggesting a connection between oral and cardiovascular diseases ^21^^,^^22^. This correlation likely arises from chronic inflammatory processes and the dissemination of oral bacteria into the bloodstream ^23^. Studies indicate that periodontitis is an independent risk factor for cardiovascular diseases, being associated with atherosclerosis and contributing to adverse cardiovascular events ^21^.

Common risk factors for both cardiovascular and periodontal diseases have already been established, such as diabetes, smoking, and serum levels of C-reactive protein ^24^. Additionally, other factors-such as hyperlipidemia, hypertension, and obesity-are also risk factors for cardiovascular diseases and appear to influence periodontal diseases ^21^^,^^22^. These factors may mediate the correlation between tooth loss and cardiovascular diseases.

These findings reinforce the idea that edentulism is not only an outcome of cumulative oral health problems but also a relevant marker of systemic vulnerability. A systematic review showed that tooth loss is associated with other higher morbidity and mortality, particularly among individuals with diabetes-a condition that amplifies systemic inflammation and metabolic dysfunction, mechanisms also linked to cardiovascular disease pathophysiology ^25^. The present study showed a significant correlation between cardiovascular disease with functional limitation and tooth loss, which aligns with emerging evidence from longitudinal studies indicating that reductions in dentition are linked to poorer cardiovascular outcomes. Another systematic review of 12 cohort studies demonstrated that individuals with tooth loss-either edentulous or with fewer than 10 remaining teeth-present a significantly higher hazard ratio (HR) for cardiovascular disease mortality (HR: 1.66; 95%CI: 1.32-2.09). This supports the view that tooth loss may operate as an indicator of systemic vulnerability, potentially mediated by chronic inflammatory processes, impaired dietary patterns, and alterations in the oral microbiome. It is reinforced the importance of incorporating tooth loss as a relevant clinical parameter within cardiovascular risk assessment and ongoing patient monitoring ^26^.

As reported in previous studies, the present results also show some influence of social determinants, evidenced by the correlation with lower HDI, suggesting that structural inequalities shape cardiovascular diseases ^27^^-^^30^. Although the eco-infodemiological study design limits causal inference, the consistency across biological mechanisms, social determinants, and epidemiological evidence strengthens the plausibility for this correlation, highlighting the need for integrated policies that expand primary oral health care coverage and consider tooth loss as a risk stratification factor for cardiovascular disease ^31^^,^^32^.

Individuals in socioeconomically disadvantaged contexts may have less access to preventive and restorative dental care, leading to higher prevalence of tooth loss. These inequalities may increase cardiovascular risk, as tooth loss and poor oral health are contributing factors to atherosclerosis and other cardiovascular complications. Public policies aimed at reducing these disparities-by improving access to dental care and promoting oral health-are essential for mitigating cardiovascular risks in vulnerable populations ^33^^,^^34^.

Several limitations of this study should be acknowledged. First, the ecological design used Brazilian states as the unit of analysis, which precludes causal inference and individual-level conclusions; the correlations observed at the aggregate level may not reflect relationships at the individual level, a phenomenon known as the ecological fallacy. Second, both tooth loss and cardiovascular disease diagnoses were based on self-reported data from the PNS 2019, which are subject to recall bias and potential underreporting, particularly among populations with limited access to healthcare services. Third, despite the inclusion of socioeconomic indicators, such as HDI and the Gini index, as contextual variables, residual confounding by well-established shared risk factors - including smoking, diabetes mellitus, hypertension, obesity, and dietary patterns - could not be fully controlled in the primary correlation analyses. Fourth, the restricted number of analytical units (n = 27, including all Brazilian states and the Federal District) limits statistical power and may affect the stability of the correlation coefficients. Fifth, the data were collected in 2019, which is the most recent population-based Brazilian study, but may not capture more recent changes in the oral health and cardiovascular profiles of the Brazilian population, particularly those occurring in the aftermath of the COVID-19 pandemic. 

Finally, tooth loss is a heterogeneous indicator that does not distinguish between the underlying etiologies - such as dental caries, periodontitis, or elective extraction - each of which may carry distinct systemic inflammatory implications, thereby limiting the specificity of the exposure definition used in this study. Further studies should continue to explore these associations and evaluate the effectiveness of policy interventions aimed at improving both oral and cardiovascular health ^34^.

## CONCLUSIONS

The findings of this eco-infodemiological study indicate that edentulism shows correlations with self-reported cardiovascular diseases in the Brazilian population. Tooth loss also demonstrated moderate positive correlation with cardiovascular disease accompanied by functional limitations or a history of surgery, suggesting that cumulative oral disease burden may reflect broader systemic vulnerability. In addition, socioeconomic indicators such as HDI and the Gini index showed strong correlations with cardiovascular outcomes.
